# The impact of sensorimotor training on physical fitness in older women with diabetes: a pilot study

**DOI:** 10.1186/s12877-025-06591-4

**Published:** 2025-11-24

**Authors:** Carolina A. Cabo, Jose A. Parraca, Orlando Fernandes, Paulo Nunes, Cláudia Mendes, Mário C. Espada

**Affiliations:** 1 Departamento de Desporto e Saúde, Escola de Saúde e Desenvolvimento Humano, Universidade de Évora, Largo dos Colegiais 2, Évora, 7000-645 Portugal; 2 Comprehensive Health Research Centre (CHRC), Universidade de Évora, Largo dos Colegiais 2, Évora, 7000-645 Portugal; 3 Escola Superior de Educação, Instituto Politécnico de Setúbal, Setúbal, 2914-504 Portugal; 4 Sport Physical activity and health research & Innovation CenTer (SPRINT), Rio Maior, 2040- 413 Portugal; 5 Life Quality Research Centre (CIEQV-Setúbal), Instituto Politécnico de Setúbal, Setúbal, 2914-504 Portugal; 6 Centro de Estudos sobre África e Desenvolvimento , CE-sA-CSG-ISEG-ULisboa, Lisboa, Portugal; 7 Universidade Lusófona’s Research Center for Biosciences and Health Technologies (CBIOS), Lisbon, Portugal; 8 Faculdade de Motricidade Humana, CIPER, Universidade de Lisboa, Lisboa, 1499-002 Portugal

**Keywords:** Exercise therapy, Proprioception, Aging, Diabetes mellitus, type 2, Range of motion, articular, Muscle strength

## Abstract

Aging and diabetes mellitus (DM) are associated with declines in physical fitness, such as reduced muscle strength, balance, and flexibility, increasing the risk of falls and functional limitations. This pilot study used a quasi-experimental design with pre- and post-intervention assessments to evaluate the effects of a 6-month sensorimotor training (SMT) program on physical fitness in older women with type 2 diabetes. Ten women aged 65 and older were divided into an intervention group (IG, *n* = 5) and a control group (CG, *n* = 5). The IG participated in a twice-weekly SMT program, while the CG maintained their usual routines. Physical fitness was assessed at baseline and after 24 weeks using the Timed Up and Go (TUG) test for agility and dynamic balance; the 30-second sit-to-stand test for lower limb strength; the arm curl test for upper limb strength; and the sit-and-reach and back scratch tests for lower and upper limb flexibility, respectively. The IG showed significant improvements in agility (TUG, *p* = 0.020), lower limb flexibility (sit-and-reach, *p* = 0.049), and upper limb flexibility on the left side (back scratch, *p* = 0.023) compared to the CG. Both groups improved in lower limb strength (*p* = 0.330), while upper limb strength showed no significant changes (*p* = 0.166). Although the CG also improved in some measures, the IG achieved greater functional gains. These results suggest that SMT enhances neuromuscular control, proprioception, and flexibility—key factors in reducing fall risk and improving independence in older adults with diabetes. SMT appears to be a promising intervention to support physical function, balance, and quality of life in this population.

**Trial registration**

Clinical trial registry 01/09/2021. Clinical trial number: NCT05398354. Trial Name: Active Retirement: Effects of the Application of a Training Program.

**Supplementary Information:**

The online version contains supplementary material available at 10.1186/s12877-025-06591-4.

## Introduction

Aging is a multifactorial process characterized as a natural and physiological process that progressively accompanies the life cycle. In addition to complications within the psychosocial domain, significant physical deterioration also occurs, resulting in cognitive slowing, depression, functional incapacity, diminished resilience, inactivity, and physical degeneration [1]. The functional changes intrinsic to the aging process, such as decreased mobility and muscle strength, may precipitate conditions of imbalance and, consequently, elevate the risk of falls among elderly individuals. Falls in the elderly population are associated not only with the use of medication but also with factors such as frailty and functional decline [2, 3].

The World Health Organization (WHO) states that there is clear evidence that people over 65 years of age who are inactive have several associated diseases and that mortality rates from strokes, coronary heart disease, type 2 diabetes mellitus (T2DM), and hypertension, among others, are higher [4, 5]. T2DM is a growing global health concern, with prevalence expected to triple in the coming decades, particularly affecting older women, who often experience worse metabolic control and greater physical limitations. DM increases the risk of cardiovascular disease, neuropathy, and musculoskeletal impairments. Age-related declines in muscle strength, balance, and coordination, combined with diabetes-related complications such as peripheral neuropathy, heighten the risk of falls and functional limitations in this population [6]. In the coming decades, the number of patients with DM is expected to triple [7].

The WHO states that there is clear evidence that regular physical activity (PA) yields significant benefits for individuals over 65 years of age. These individuals are usually less active and, therefore have diseases related to inactivity, such as DM. This metabolic disease mortality rates are lower among older adults who are more active than among those who are less active [8].

Despite these known benefits, fewer than one-fifth of people with diabetes meet recommended physical activity levels [9]. Exercise is widely recognized as a cornerstone of diabetes management, offering significant benefits for glycemic control, cardiovascular health, and overall well-being. Regular PA has been shown to improve insulin sensitivity, reduce HbA1c levels, and support weight management, which are critical for older women with diabetes. Additionally, exercise enhances muscle strength, bone density, and balance, reducing the risk of falls and osteoporosis, which are common concerns in aging populations. Psychological benefits, including reduced stress and improved mental health, also contribute to a better quality of life for older women managing diabetes [10, 11]. Furthermore, exercise reduces total daily insulin requirements, stress, and depression while improving the quality of life (QoL) of individuals [12–14].

Recent studies underscore the critical role of education in the self-management of type 2 diabetes, particularly among older adults. A qualitative and quantitative study conducted in Indonesia demonstrated that the implementation of a manual self-management companion book was highly accepted by diabetic patients and healthcare providers alike. Most participants—elderly individuals from lower socioeconomic backgrounds—lacked access to digital devices, reinforcing the value of printed educational materials. The manual book served not only as an informational tool but also as a monitoring aid, allowing patients and health professionals to track glycemic levels, HbA1c results, and medication use over time. These findings support the idea that accessible, culturally adapted educational resources are essential for enhancing patients’ understanding, compliance, and quality of life [15].

A systematic literature review identified eight core elements of precision health care for patients with diabetes: self-management, interdisciplinary collaborative practice, personalized genetic or lifestyle factors, glycemic targets, patient preferences, glycemic control, patient priority–directed care, and biodata- or evidence-based practice. These components form a comprehensive framework for implementing precision healthcare strategies in clinical settings, offering a structured and patient-centered approach to diabetes care [16].

Sensorimotor training (SMT) was selected for this study due to its unique focus on improving balance, proprioception, and neuromuscular coordination—all critical areas that decline with age and are further compromised by diabetes. Unlike traditional strength or aerobic exercise, which typically focuses on cardiovascular health or muscle strength, SMT directly engages neural and sensory systems essential for physical stability, coordination, and efficient movement [17]. With age and in the context of diabetes, proprioceptive feedback deteriorates due to peripheral neuropathy and neurodegeneration, which compromises balance and increases fall risk [18, 19]. The SMT stimulates the neuromuscular system by activating sensory pathways and motor responses that maintain postural control. This approach is essential for addressing age-specific and diabetes-related declines in physical function [20].

Neuroplasticity is a key mechanism through which SMT exerts its effects. Repetitive sensorimotor stimuli help restore proprioceptive feedback by stimulating mechanoreceptors and strengthening neural circuits involved in postural control and coordination [21]. These adaptations improve reaction times and dynamic stability, contributing to fall prevention and better mobility in this population.

Skeletal muscle function also benefits from SMT. By activating both slow- and fast-twitch fibers, sensorimotor exercises improve muscular endurance and power, combatting age- and diabetes-related muscle atrophy [22]. Neuromuscular adaptations enhance motor unit synchronization, enabling more efficient movement execution. These physiological effects likely explain the improvements observed in agility and lower limb strength among participants in the intervention group. Increased muscle strength is essential for preserving autonomy and reducing fatigue in daily life [23].

This specific training focuses on improving neuromuscular control, postural stability, and coordination by challenging the body’s sensory and motor systems. Research has shown that SMT improves gait, muscle activation, and overall balance in older adults with T2DM [24, 25]. Given that older women with diabetes face higher risks of functional decline, loss of independence, and lower quality of life compared to men [26], implementing SMT as a targeted intervention could offer significant physical and psychological benefits.

Therefore, this study aimed to evaluate the effects of a 6-month SMT program on physical fitness in older women with type 2 diabetes mellitus.

## Materials and methods

### Design

This investigation is part of a study protocol, registered at ClinicalTrials.gov (Identifier: NCT05398354) under the title “Active Retirement: Effects of the Application of a Training Program.” The authors confirm that all ongoing and related trials for this intervention are registered. This is a quasi-experimental, two-arm pilot study with pre- and post-intervention assessments. Participant recruitment occurred from 1 November 2021 to 2 January 2022. This study was developed at the University of Évora, and the measurements were made at two time points: the first on 5 January 2022 and the second on 30 July 2022, both in the municipality of Almada (Portugal).

### Participants

**Fig. 1:**
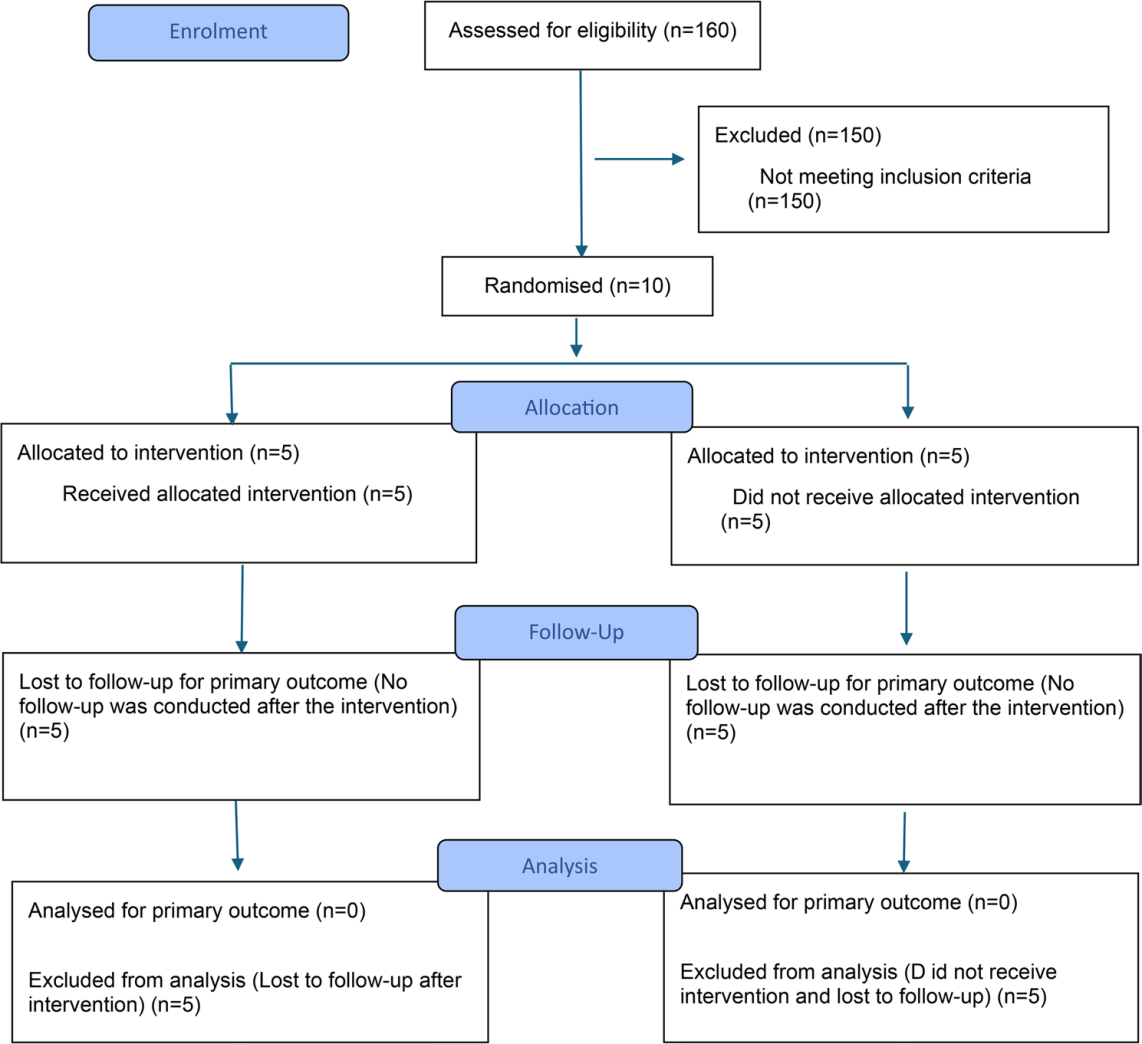
CONSORT 2025 flow diagram

Ten elderly women with type 2 DM were selected from the list of registered clinical trials to participate in this pilot study. These 10 women were part of an intervention and control group (IG and CG, respectively) comprising a total of 160 older people. Detailed information about treating diabetic patients typically involves understanding the different approaches tailored to individual needs, including lifestyle modifications, oral anti-diabetic drugs, and insulin administration. In this study, participants were treated with insulin (Fig.1).

Subjects were selected because they have similar characteristics (weight, height, and body mass index - BMI), apart from age, and both suffer from the same pathology DM. All the samples were evaluated at equivalent times. Thus, we analyzed five participants in the IG, who carried out the training program in addition to the assessments, and a second group, the CG, who carried out only the initial and final assessments without having practiced the training program. The selection of participants was random. The inclusion criteria were: (1) women over 65 years of age; (2) diagnosis of type 2 diabetes mellitus; (3) not having undergone recent (< 6 months) surgical intervention; (4) no musculoskeletal diagnosis or problems with locomotion; (5) no psychiatric or neurological disorders; and (6) no clinical cardiovascular conditions. Exclusion criteria included: (a) presence of vascular complications related to diabetes—including nephropathy, retinopathy, or polyneuropathy (due to safety concerns and to maintain homogeneity in this initial pilot sample, as neuropathy can significantly alter balance and proprioceptive function); (b) high arterial hypertension; (c) diabetic foot; and (d) other diseases affecting the lower limb’s function.

### Intervention

The intervention group participated in a 24-week SMT program, delivered twice weekly (Frequency), with each session lasting 45 min (Time). The training intensity was progressive (Intensity) and based on each participant’s tolerance and physical condition. The Type of exercise focused on balance, coordination, proprioception, and neuromuscular control. This structure followed the FITT principle (Frequency, Intensity, Time, and Type), a recognized model for prescribing individualized and progressive training programs [27]. The program was structured into three progressive intensity phases:

- Weeks 1–8 (Initial Phase – Easy): Exercises without external resistance, focusing on postural control, balance, and functional movement.
- Weeks 9–16 (Intermediate Phase): Introduction of low-level resistance using elastic bands, ankle weights, and hand-held weights.
- Weeks 17–24 (Advanced Phase): Further increase in load intensity using the same equipment to promote strength and neuromuscular control.

Each session followed a consistent structure:

- Warm-up (10 min): Light walking followed by joint mobility exercises.
- Main training (25 min): Circuit training composed of 4 rounds of 8 exercises (50 s of activity, 15 s of rest). Exercises targeted balance, coordination, strength, and proprioceptive stimulation.
- Cool-down (10 min): Static stretching and breathing exercises.

Progression was individualized based on performance and tolerance. Post-session perceived exertion was recorded using the Borg Scale, and monthly enjoyment and satisfaction were monitored through the Physical Activity Enjoyment Scale (PACES). Attendance was recorded at every session.

The control group did not receive any structured physical training and continued with their usual activities. They only participated in the baseline and final assessments.

### Ethics approval

The Ethics Committee of the University of Évora approved this project (approval number: 21040). In addition, the participants provided written informed consent following the Helsinki Declaration [28].

### Sample size

Sample size calculations were performed using the G*Power 3.1.9.4 software (Kiel University, Kiel, Germany), selecting the statistical test to compare the difference between two independent means (two groups). The calculation was based on a power of 0.80, α = 0.05, and an estimated moderate-to-large effect size (Cohen’s d = 0.99), which indicated a minimum of 5 participants per group for this pilot study. The participants were selected to identify clinically relevant differences in physical fitness outcomes.

### Instruments

#### Anthropometry and body mass index

Body weight (scale), height (stadimeter), and BMI were assessed. Before beginning any of the measurements, the participants were asked to remove their shoes, socks, and heavy clothing (jackets, sweaters, coats, etc.). They were also asked to empty their pockets and remove their belts and other accessories (bands, pendants, etc.). Height was measured via a stadiometer (Seca 22, Hamburg, Germany). The instrument was placed on a vertical surface with the measuring scale perpendicular to the ground. The participants stood with their shoulders balanced and their arms relaxed along the body. Height was measured in cm and rounded to the nearest mm. Body weight was measured on a scale. Body weight was recorded in kg. BMI was determined via the following formula: weight/height2.

#### Physical fitness

To assess physical fitness, two attempts were made for each test applied, including the TUG, strength, and flexibility tests. All instruments and tests used in this study (e.g., TUG, sit-to-stand, arm curl, sit-and-reach, and back scratch) are validated and widely used in older adult populations. Their reliability has been demonstrated in previous studies, with test-retest intraclass correlation coefficients (ICCs) typically ranging from 0.80 to 0.97. Between the two attempts, participants were given a 2-minute rest.The following measurements were then taken:

I. Agility and speed were assessed using the Timed Up and Go (TUG) test in seconds (s), in which participants were instructed to stand up from a standard chair, walk 3 m, turn around, walk back, and sit down again [29];
II. (ii and iii) Strength was evaluated through the number of repetitions (reps) performed from two tests. The first consisted of evaluating lower limb strength (SL) by counting how many times the participant could sit down and stand up from a chair for 30 s [30], and the second upper limb strength was determined by the number of times a weight could be lifted by performing flexion-extension of the arms (FA) for 30 s [30];
III. (iv and v) flexibility was evaluated via two tests. For flexibility of the lower limbs, a “sit and reach” (SA) was performed, where the participants, from a sitting position with one leg extended, slowly lowered themselves, sliding their hands down the extended leg until touching (or passing) their toes [30]. For flexibility of the upper limbs, the “behind the back reach right and left” tests (AD and AE) were carried out, which evaluated the entire range of movement of the shoulder and consisted of measuring the distance between (or the overlap of) the middle fingers behind the back with a ruler [30]. In both cases, the measurement used was in centimeters (cm).

### Procedures

A variety of tools were used for the assessments, based on the tests applied. All measures were taken at the beginning and end of the intervention. Before the first measurement, all the participants went through a familiarization phase to familiarize themselves with the different instruments and assessments included in this project.

**Fig. 2:**
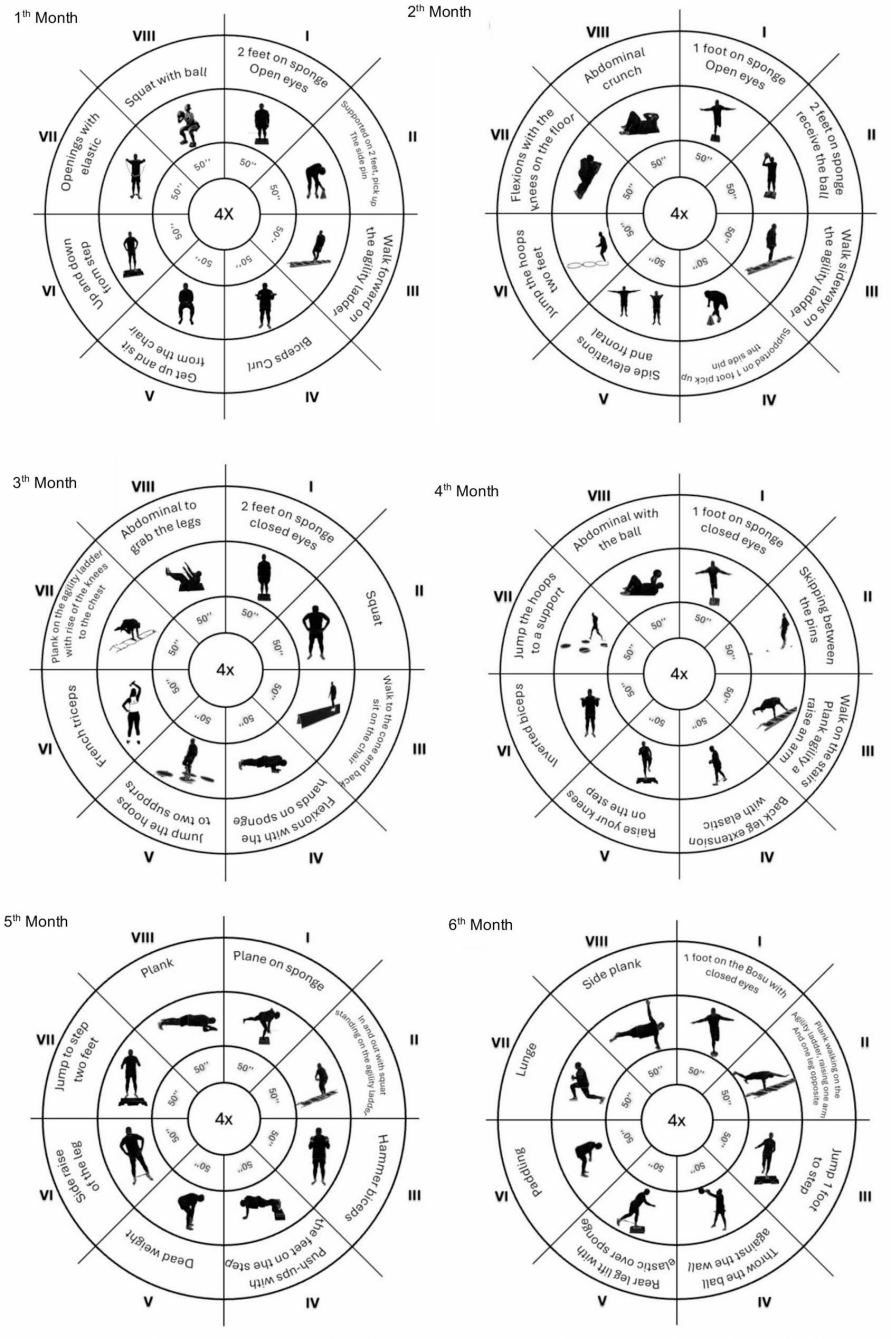
Intervention used for sensorimotor training [32]

Experimental group: The participants enrolled in the SMT program carried out exercises for 6 months, twice a week. As the schedule progressed, the load progressively increased. To this end, the session was divided into three levels of intensity: easy (no external load during the first eight weeks), intermediate (application of an external load: elastic bands, shin guards, and free weights, from the 9th to the 16th week) and advanced (increase in the external load to the previous level, from the 17th to the 24th week). Each month, a different type of session was developed. The duration of each session was 45 min, which was divided into three phases: the initial phase (10 min), consisting of 5 min of walking followed by a joint warm-up; and the fundamental phase (25 min), where we worked on an exercise circuit. This circuit consisted of 4 cycles, with eight exercises each (50s on, 15 s off) and a return to calm (10 min), where muscle stretching was performed. In addition, at the end of each session, the intensity was evaluated via the perceived exertion (PSE) scale, and the adherence rate and satisfaction level were measured via the Physical Activity Enjoyment Scale (PACES) [31]. In the CG, the participants continued with their normal daily routine, with only participants in the assessments (Fig.2).

### Statistical analysis

Descriptive statistics and calculations were performed using Jamovi (version 1.6) [computer software]. The data are presented as the means and standard deviations (M ± SD). We tested for normality using the Shapiro-Wilk test and for homogeneity using Levene’s test. Parametric or nonparametric tests were performed depending on data distribution.

To compare the dependent variables, Student’s t-test for paired samples was used within groups, and two-way ANOVA (group × time) was applied after test for sphericity and homogeneity of variances to evaluate between-group differences. Statistical significance was set at *p* < 0.05. The effect size (ES) was used to analyze the magnitude of change and was interpreted according to Eta squared (η²): Small effect size (0.01–0.06); Medium effect size (0.06–0.14); Large effect size (> 0.14).

## Results

### Participant details

Table 1 shows the details of the study participants. According to the results, these ten women were similar in age. In terms of weight and height, they are similar, both being overweight [33].

**Table 1:** Baseline characteristics of the participants

Group/Variables	CG	IG	*p*
*Age* *(years)*	80.00 ± 3.54	74.60 ± 3.78	0.048
*Weight* *(kg)*	72.80 ± 19.90	74.90 ± 14.80	0.856
*Height* *(m)*	1.53 ± 0.05	1.54 ± 0.06	0.719
*BMI* *(kg/m*^*2*^*)*	31.20 ± 8.20	31.60 ± 6.16	0.937

The Student’s t values indicate no significant differences between groups, except for age, which was significantly different (*p* = 0.048).

To assess physical fitness, the following measurements were carried out: agility and speed of execution; strength; and flexibility. Both the weight and BMI of both groups decreased, with the IG having a greater reduction.

With regard to agility and speed, as shown in Table 2, the CG, despite having an initial value of 9.26s, which is greater than that of the IG, managed to improve its test execution time after the intervention to 7.59s. The IG started at 8.46s and reached 7.49s in the second evaluation.

**Table 2:** Main outcomes between the first and second assessments in both groups (CG *n* = 5; IG *n* = 5)

Variables	Before Exercise Baseline		After Exercise 24 weeks		*p*	η^2^	CI 95%
	CG	IG	CG	IG			
	M ± SD	M ± SD	M ± SD	M ± SD			
*Weight* *(kg)*	72.80 ± 19.90	74.90 ± 14.80	71.50 ± 19.10	72.30 ± 15,4	*p* = 0.351	0.109^#^	(54.1;91.6)
*BMI* *(kg/m*^*2*^*)*	31.20 ± 8.20	31.60 ± 6.16	30.60 ± 7.79	30.50 ± 6,63	*p =* 0.406	0.088^#^	(23.4;38.5)
*TUG* *(s)*	9.26 ± 2.38	8.46 ± 1.36	7.80 ± 1.63	7.49 ± 0.80	*p* = 0.649	0.027^#^	(7.1;9.5)
*SL* *(rep)*	11.60 ± 2.70	12.20 ± 1.92	13.8 ± 4.09	13.20 ± 1.30	*p* = 0.330	0.118^#^	(10.1;15.3)
*FA* *(rep)*	16.80 ± 3.03	18.80 ± 5.22	18.20 ± 3.56	18.20 ± 4.38	*p* = 0.166	0.225^#^	(13.4;22.6)
*SA* *(cm)*	−6.80 ± 14.40	−4.40 ± 9.18	−1.60 ± 9.48	−0.20 ± 8.98	*p =* 0.719	0.017^+^	(−15.1;8.6)
*AD* *(cm)*	−23.30 ± 18.00	−17.60 ± 15.30	−20.20 ± 17.90	−10.20 ± 11.10	*p* = 0.290	0.138^#^	(−37.8;−5.7)
*AE* *(cm)*	−28.40 ± 13.30	−19.60 ± 14.30	−35.00 ± 18.80	−13.60 ± 11.70	*p* = 0.083	0.329*	(−46;−2.3)

Data presented as mean ± *SD* standard deviation

*CG *Control Group, *IG *Intervention Group, *BMI* body mass index, *TUG *timed up and go test, *SL *sit and stand test, *FA *forearm flexion test, *SA *sit and reach test, *AD *reach behind the back test–right, *AE *reach behind the back test–left

ANOVA was used to assess the global group effect at all time points. The post hoc test revealed results within each group; a *p* value <0.05 indicated statistical significance

^+^Small effect size (0.01-0.06); ^#^ Medium effect size (0.06-0.14); * Large effect size (>0.14)

In terms of strength capacity, the strength of the lower limbs was evaluated through the sit-and-stand test (SL), and the strength of the upper limbs was evaluated through the forearm flexion test (FA). In the SL test, the CG had a lower mean value in the first evaluation (11.60 repetitions), and in the second evaluation, it increased the mean value to 13.80 repetitions. The IG started with a slightly greater value of 12.20 repetitions and then increased one more repetition to 13.20 repetitions in the second evaluation.

Table 2 shows that CG in the FA test resulted from the first evaluation of 16.80 repetitions, but in the second evaluation, the result increased by two repetitions to 18.20 repetitions. The IG obtained a superior result in the first assessment of 18.80 repetitions but reduced its value to 18.20 repetitions, finishing the second with the same number of reps as the CG. Although we do not have significant *p* values, we have some moderate ES, such as weight (*p* = 0.351), BMI (*p* = 0.406), SL (*p* = 0.330), FA (*p* = 0.166), AD (*p* = 0.290), and large ones, such as TUG (*p* = 0.649) and SA (*p* = 0.719). Because our objective was to understand the impact of exercise on these patients, as shown in Table 2, the IG improved, as did the CG. In other words, the group that received the in-tervention exhibited substantial differences even though there were no significant differences between the groups, which supports the ES that we previously reported.

In terms of flexibility, we analyzed the flexibility of the lower limbs through the sit and reach test (SA) and the flexibility of the upper limbs through the test reach behind the right back (AD) and reach behind the left back (AE). As we can see in Table 2, in the SA test, CG obtained a value of −6.80 cm in the first evaluation, which was worse than that of IG (−4.40 cm), but its result improved by five cm, reaching − 1.60 cm. On the other hand, the IG ranged from − 4.40 cm in the first assessment to −0.20 cm. Both groups improved their scores on average in this test. In the AD test, CG presented an initial value of −23.30 cm, which was worse than that of IG (−17.60 cm) but presented improvements of three cm for the second evaluation (−20.20 cm), whereas IG improved its result by seven cm, ranging from − 17.60 cm on average to −10.20 cm. In this case, the IG obtained better results. In the AE test, CG has an initial value of −28.40 cm, which is greater than that of IG (−19.60 cm), and its value increases from 7.00 cm to −35.00 cm. The IG, in addition to presenting a lower initial value, reduces the distance on average by 5.00 cm, ending the second evaluation with a value of −13.60 cm.

To observe the distinct evolution of individuals who underwent intervention and those who did not, we performed a statistical analysis of the variation. We confirmed that there were significant differences in the results of the TUG (*p* = 0.020), SA (*p* = 0.049), and AE (*p* = 0.023) tests, as shown in Table 3.

**Table 3:** Comparative analysis of variation after the exercise program (CG *n* = 5; IG *n* = 5)

Variables		CG		IG
	24-weeks	*p* value	24-weeks	*p* value
*TUG* *(s)*	1.46	0.221	0.97	0.020
*SL* *(rep)*	−2.20	0.051	−1.00	0.298
*FA* *(rep)*	−1.40	0.052	0.40	0.646
*SA* *(cm)*	5.20	0.079	4.20	0.049
*AD* *(cm)*	3.10	0.212	7.4	0.080
*AE* *(cm)*	−6.60	0.343	6.00	0.023

*CG *Control Group, *IG *Intervention Group, *AV1 *1 st assessment, *AV2 *2nd assessment, *TUG *timed up-and-go test, *SL *sit-and-stand test, *FA *forearm flexion test, *SA *sit-and-reach test, *AD* reach behind the back test–right, *AE *reach behind the back test–left

*p* value: *p* < 0.05 shows significant differences

## Discussion

This research aimed to analyze the effects of Sensorimotor Training (SMT) on physical fitness throughout the aging process in diabetic women. The main findings indicate that the intervention group (IG) experienced significant improvements in agility, speed (assessed by the TUG test), lower limb strength, and flexibility compared to the control group (CG), while upper limb strength showed inconsistent changes. These results suggest that SMT enhances neuromuscular control, postural stability, and functional independence, which are critical factors for fall prevention in this population. Our findings align with previous research indicating that sensorimotor exercises improve neuromuscular control and proprioception, thereby reducing fall risk in older adults [34, 35]. However, unlike some studies that reported consistent improvements in upper limb strength, our results showed a decrease in upper limb strength repetitions in the IG, which may be attributed to the SMT program’s primary focus on lower extremity function and postural control. This specificity highlights the importance of tailoring interventions to target both upper and lower body components when comprehensive strength improvements are desired.

Previous studies have reported that proprioception loss is a major cause of balance deficits and falls in the elderly. Our data support this, demonstrating improved proprioceptive control following SMT. However, despite improvements in flexibility, both groups remained within lower percentiles, suggesting that additional mobility-focused exercises may be necessary to enhance this domain further. These nuanced findings contribute to the understanding of how different physical qualities respond to SMT in diabetic elderly populations. A major concern for professionals working with elderly individuals is the high number of falls. These findings are corroborated by Chandler [36], who reported that fatigue resistance can affect the ability to respond effectively to a disturbance in equilibrium, which is associated with mobility. Studies have shown that precarious mobility and a decrease in physical fitness are predictors of morbidity and mortality. Changes in mobility predict loss of independence and death in people over 65 years of age; individuals with mobility impairments have a higher risk of death and dependence than those who maintain their mobility [37]. Similarly, low levels of cardiorespiratory fitness have been associated with the risk of morbidity and mortality from chronic degenerative diseases, including arterial disease, coronary artery disease, systemic arterial hypertension, DM, and some types of cancer [38].

An experimental study with diabetic rats investigated the effects of quercetin supplementation and moderate aerobic training on myostatin and follistatin levels in heart tissue. The animals were divided into control, diabetic, diabetic with supplementation, diabetic with exercise, and diabetic with combined supplementation and exercise groups. The results showed that both quercetin supplementation and exercise alone significantly reduced myostatin levels, a protein associated with muscle growth inhibition, with the most pronounced effect observed when both interventions were combined. Additionally, follistatin levels, an antagonist of myostatin, significantly increased in all treated diabetic groups compared to the healthy control group. These findings suggest that aerobic training and quercetin supplementation may beneficially modulate proteins related to muscle and cardiac health in diabetes, potentially contributing to therapeutic strategies aimed at improving cardiovascular and muscular function in this context [39].

Aging is associated with a decline in muscle strength, particularly in the lower limbs, which contributes to mobility im-pairments and reduced independence. This study revealed that both IG and CG showed improvements in lower limb strength following the intervention. However, upper limb strength did not improve consistently, possibly due to the specificity of SMT, which primarily targets postural control and lower extremity function. Similar studies by Heubel et al.[40] found that multicomponent training, including strength and balance exercises, significantly enhanced functional fitness and glycemic control in older adults with DM. Aging results in a decline in physical qualities, including strength [1, 10, 11, 41]. When the values obtained for lower limb strength were analyzed, we found that both methods improved the results. In the upper limb strength test, the IG decreased the number of repetitions performed in the first assessment, whereas the CG increased it. According to the reference values of Baptista and Sardinha [42] in the strength test of the lower limb strength test, the CG and IG are in the 50th percentile. The upper limb strength test results of the CG and IG were between the 75th and 90th percentiles. The values obtained in the strength test prove that both participants presented excellent capacity concerning general standards for their age. Additionally, as one of the factors that contribute to im-provements in lower limb proprioception is the learning effect, a steady increase in the difficulty of sensorimotor exercises leads to increased proprioception [18].

Flexibility, particularly in the lower limbs, is crucial for maintaining functional movement patterns and preventing injuries. The results indicated greater improvements in lower limb flexibility compared to upper limb flexibility, aligning with findings from Baptista and Sardinha [42] who reported that progressive sensorimotor exercises improve range of motion and joint stability. However, despite improvements, both IG and CG remained within lower percentiles for flexibility, suggesting that additional stretching or mobility-focused exercises may be needed alongside SMT for more comprehensive benefits.

One of the key findings of this study was the improvement in proprioceptive control, which is particularly beneficial for elderly individuals with diabetes-related neuropathy. Proprioception loss is a major contributor to balance deficits and fall risk in older adults [43]. SMT, which includes unstable surface training, single-leg stance exercises, and coordinated movement drills, has been shown to improve postural stability and equilibrium control, leading to greater confidence in movement and reduced fear of falling. Heubel et al.[40] verified the effects of 16 weeks of multicomponent training, composed of strength, flexibility, and balance exercises, on the functional fitness and glycemic parameters of 13 elderly people with DM. These researchers reported significant improvements in the mean flexibility indices in the sit and reach test (11.40 ± 8.70 cm; 14.50 ± 9.80 cm) and in the strength and endurance of the upper limbs, as evaluated by the elbow flexion test at 30’’ (first evaluation 16.60 ± 3.40 reps; second evaluation 19.40 ± 4.20 reps). Compared with our study, the lower flexibility values obtained are much greater, whereas the upper limb strength values are similar. Thus, we can say that once again, the intervention improved not only the capacity for agility and speed but also the flexibility of the upper and lower limbs. Having only reduced the strength of the upper limbs.

One of the strengths of this study is its focus on a specific clinical population—elderly women with type 2 diabetes—who are at increased risk of falls and mobility impairments. By demonstrating the benefits of SMT on key functional parameters such as agility, strength, and proprioception, our findings have practical implications for clinicians and exercise professionals designing fall prevention programs. The progressive and individualized nature of the training, grounded in the FITT principle, makes it adaptable to different levels of fitness and clinical status.

Furthermore, the observed improvements in mobility and muscle function have potential indirect benefits for glycemic control and cardiovascular health, supporting SMT as a complementary intervention for diabetes management40. However, the small sample size and age differences between groups limit generalizability. Future studies with larger, age-balanced cohorts are needed to confirm these findings and optimize training protocols across diverse elderly populations [44].

A limitation of this research is the small number of participants, largely due to the strict inclusion criterion of having diabetes mellitus. This reduces statistical power and increases the risk of Type II errors, potentially obscuring subtle but clinically relevant effects. Future research should involve larger samples and explore physiological markers such as glucose metabolism to deepen understanding of SMT’s multifaceted benefits.

## Conclusion

This study highlights the positive effects of SMT on physical fitness in older women with diabetes. The observed gains in agility, strength, flexibility, and proprioception emphasize its potential for fall prevention and maintaining functional independence. Improvements in proprioception and neuroplasticity enhance balance and coordination, thereby reducing fall risk. Adaptations in skeletal muscle led to increased strength and agility, which contributed to enhanced mobility and independence. Although no metabolic biomarkers were measured in this study, improved mobility and muscle function may indirectly support better metabolic regulation and insulin sensitivity, as suggested by previous research [45]. Flexibility gains help counteract joint stiffness, promoting easier movement, while potential cardiovascular and hormonal benefits, reported in related literature, may also contribute to overall health. By addressing multiple physiological factors, SMT may emerge as an effective strategy for counteracting diabetes-related physical decline and enhancing quality of life for aging women with diabetes. The study have several limitations, despite random assignment, a baseline imbalance was observed, with age differing marginally between groups (*p* = 0.048). This represents a potential limitation, as age is a established confounding variable strongly associated with functional capacity. While we attempted to control for this statistically by including age as a covariate in our secondary analysis, residual confounding may persist. However, considering the limitations of sample size and intervention duration, future research should seek to investigate long-term effects and refine training protocols to maximize benefits for older adults with diabetes.

## Supplementary Information

Supplementary Material 1.

## Data Availability

The data presented in this study are available on request from the corresponding author.
