# Supplementary material for: The impact of sensorimotor training on physical fitness in older women with diabetes: a pilot study
Source: BMC Geriatr. 2025 Nov 24;25:944. doi: 10.1186/s12877-025-06591-4 (PMC12641995; doi:10.1186/s12877-025-06591-4)
Supplement: Supplementary file 1 — Supplementary Material 1. [file 12877_2025_6591_MOESM1_ESM.docx]

**Figures**

Analysis

Lost to follow-up for primary outcome (No follow-up was conducted after the intervention) (n=5)

Randomised (n=10)

Allocation

Follow-Up

Allocated to intervention (n=5)

Received allocated intervention (n=5)

Allocated to intervention (n=5)

Did not receive allocated intervention (n=5)

Enrolment

Assessed for eligibility (n=160)

Excluded (n=150)

Not meeting inclusion criteria (n=150)

Lost to follow-up for primary outcome (No follow-up was conducted after the intervention) (n=5)

Analysed for primary outcome (n=0)

Excluded from analysis (D id not receive intervention and lost to follow-up) (n=5)

Analysed for primary outcome (n=0)

Excluded from analysis (Lost to follow-up after intervention) (n=5)

**Figure 1.** CONSORT 2025 Flow diagram.


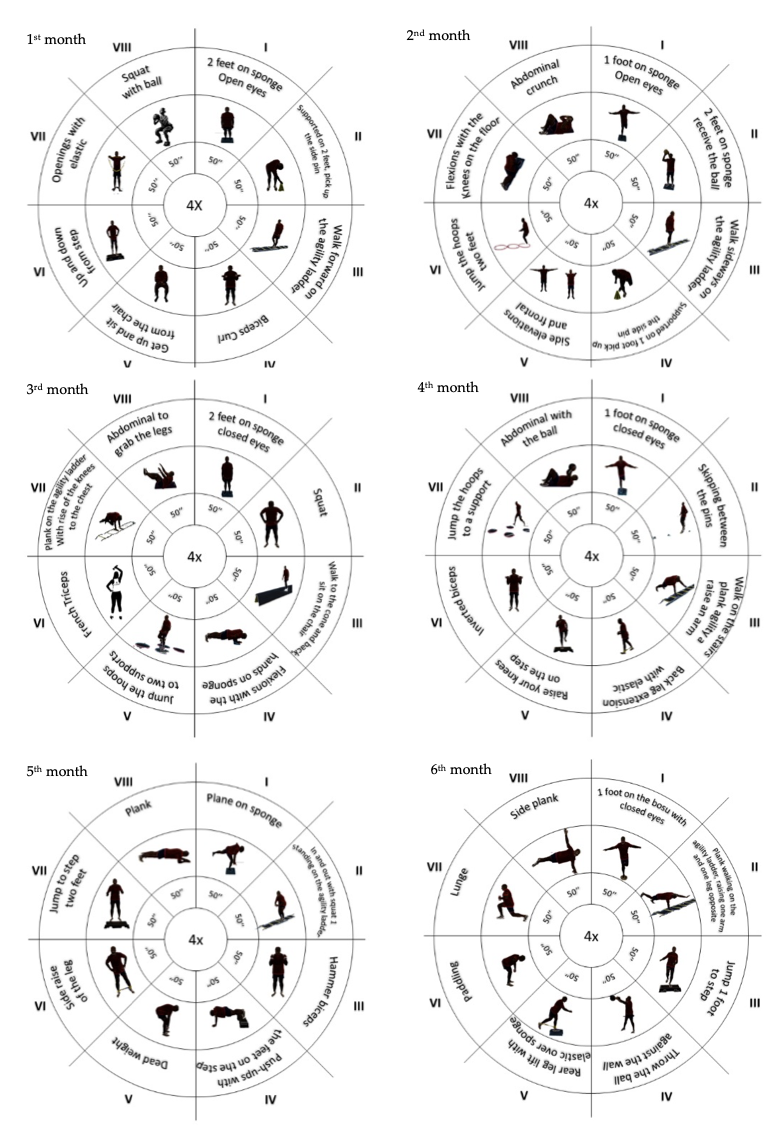


**Figure 2.** Intervention used for sensorimotor training^31^.
